# *Bathynomus vaderi* Ng, Sidabalok & Nguyen, 2025: the senior synonym of *B. paracelensis* Huang & Kawai, 2025 (Crustacea, Isopoda, Cirolanidae)

**DOI:** 10.3897/zookeys.1273.179260

**Published:** 2026-03-17

**Authors:** Peter K. L. Ng, Niel L. Bruce, Conni M. Sidabalok, Chien-Hui Yang, Thanh Son Nguyen, Shane T. Ahyong

**Affiliations:** 1 Lee Kong Chian Natural History Museum (LKCNHM), 2 Conservatory Drive, National University of Singapore, Singapore 117377, Singapore North-West University Potchefstroom South Africa; 2 Biodiversity and Geosciences Program, Queensland Museum, PO Box: 3300, South Brisbane BC, Queensland 4101, Australia National University of Singapore Singapore Singapore; 3 Water Research Group, Unit for Environmental Sciences and Management, North-West University, Private Bag X6001, Potchefstroom 2520, South Africa Biodiversity and Geosciences Program, Queensland Museum Queensland Australia; 4 Research Center for Biosystematics and Evolution, National Research and Innovation Agency (BRIN), KST Soekarno, Jalan Raya Jakarta Bogor Km 46, Cibinong 16911, Indonesia National Taiwan Ocean University Keelung Taiwan; 5 Institute of Marine Biology and Center of Excellence for the Oceans, National Taiwan Ocean University, Keelung 202301, R.O.C, Taiwan National Research and Innovation Agency (BRIN), KST Soekarno Cibinong Indonesia; 6 Department of Applied Zoology, Faculty of Biology, VNU University of Science, Vietnam National University, Ha Noi, 334 Nguyen Trai, Thanh Xuan, Hanoi, Vietnam Vietnam National University Hanoi Vietnam; 7 Australian Museum Research Institute, Sydney, NSW 2010, Australia, and School of Biological, Earth & Environmental Sciences, University of New South Wales, Kensington, NSW 2052, Australia University of New South Wales Kensington Australia

**Keywords:** Description, genetic, holotype, morphology, South China Sea, supergiant

## Abstract

*Bathynomus paracelensis* Huang & Kawai, 2025 was recently described based on four female specimens from the Paracel Islands in the South China Sea, and distinguished from *B. vaderi* Ng, Sidabalok & Nguyen, 2025 from Vietnam, by 14 morphological characters, with the primary ones being the body size and shape, pleotelson—size ratio and structure of the spines, as well as the numbers of uropod robust setae and their shape. Morphological comparisons of the type specimens of both species show that none of these characters are valid, with the two species also share nearly identical COI and 16S mitochondrial DNA sequences. As such, *B paracelensis* is here regarded as a junior synonym of *B vaderi*.

## Introduction

The cirolanid genus *Bathynomus* A. Milne-Edwards, 1879 continues to attract considerable attention, with more than 11 research articles have been published since 2000 and 10 new species (seven extant and three fossils) described since the review by Lowry and Dempsey (2006) (Shipley et al. 2016; Kou et al. 2017; Hyžný et al. 2020; Sidabalok et al. 2020; Huang et al. 2022; Ahyong 2025; Huang and Kawai 2025; Ng et al. 2025). Owing to their large size, the genus garners the interest of researchers who are not primarily focused on cirolanid taxonomy, or indeed isopod or crustacean taxonomy altogether, increasing the opportunity for misidentifications and the perpetuation of incorrect species distribution records. We cite one such as an example only, that of Ardura et al. (2021) which incorrectly reported *B. giganteus* A. Milne-Edwards, 1879 as a “non-native” central Pacific species with a “native range” in the North Pacific (*B. giganteus* occurs only in the western Atlantic). As Ahyong (2025: 170) pointed out in relation to misidentifications by Sankar et al. (2011: 113) and Prasanna Kumar et al. (2020: 1) of Indian *Bathynomus*: “Unfortunately, such instances serve to underestimate actual species diversity and hinder the growth in taxonomic knowledge of the genus.”

*Bathynomus* comprises two groups of species, which are primarily separated by size and termed “giants” and “supergiants” (Lowry and Dempsey 2006). This is similar to members of the *Cirolana* “*parva*-group”, where species are distinguished and identified by differences shown by combination of four or five characters but often further supported by other more subtle characters (e.g. see Bruce 2004; Sidabalok and Bruce 2017). This being the case, it is imperative that fully detailed descriptions be given of all the characters used for a standard detailed cirolanid species description. It is also critically important that the variation in characters is fully documented and quantified, particularly in relation to the extent and number of uropodal setae and pleotelson spines. The case presented here demonstrates the need to fully document such variation as well as the value of molecular data in confirming species identities.

A new species of supergiant isopod, *B. vaderi*, was described by Ng et al. (2025) from Vietnam off Quy Nhơn, precise position not recorded (published in January 2025). Huang and Kawai (2025) subsequently described a new species, *B. paracelensis*, supposedly from the Paracel Islands (= Xisha Islands, Hoàng Sa Archipelago) collected by Taiwanese fishermen (published in March 2025).

Huang and Kawai (2025) argued that *B. paracelensis* differed from *B. vaderi* in more than a dozen morphological characters that justified recognising it as a separate species. Huang and Kawai (2025) provided genetic data for *B. paracelensis* but commented that the absence of similar information for *B. vaderi* did not allow for comparisons, even though tissues of *B. vaderi* had already been given to Huang in June 2024 by the first author. Given that the two nominal species are morphologically closest to each other and their respective type localities are adjacent in the northern part of the South China Sea, there is an obvious question of whether they are conspecific. We herein re-evaluate the taxonomic status of *B. paracelensis* with respect to *B. vaderi* based on re-study of the type material and comparison of molecular data of both nominal species.

## Material and methods

Specimens examined are deposited in the National Museum of Marine Biology—CrustaceaDecapoda, National Museum of Marine Biology and Aquarium, Taiwan (NMMB); Zoological Reference Collection of the Lee Kong Chian Natural History Museum (previously the Raffles Museum of Biodiversity Research), National University of Singapore (ZRC); Australian Museum, Sydney (AM); Muséum National d’Histoire Naturelle, Paris, France (MNHN); Museum Zoologicum Bogoriense, BRIN, Cibinong, Indonesia (MZB); Taiwan National Museum, Taipei, Taiwan (TMCD); crustacean collection of the National Taiwan Ocean University, Keelung, Taiwan (NTOU); and Zoology Collection of the Biological Museum, VNU University of Science, Hanoi, Vietnam (ZVNU). The terminology used follows Bruce (2009), Keable (2006) and Lowry and Dempsey (2006). Measurements, in millimetres, provided are made of the maximum total length (TL), measured longitudinally from the base of the rostrum to the base of the pleotelson spines. The measurements of the length and width of the anterior part of the clypeus follows Fig. 3A.

**Figure 1.**
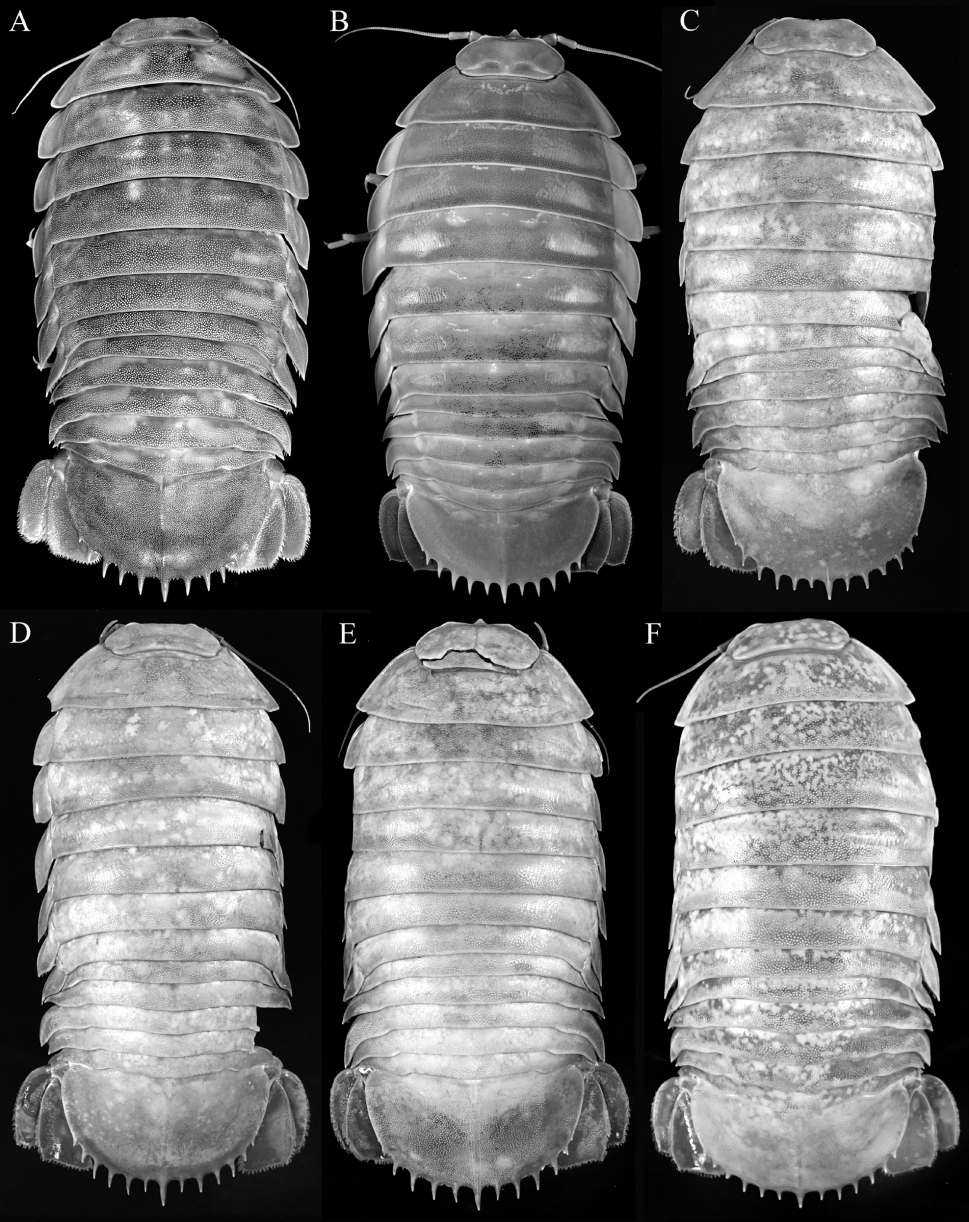
*Bathynomus vaderi* Ng, Sidabalok & Nguyen, 2025, dorsal habitus. **A**. Holotype male (TL 266 mm) (ZRC 2022.0621); **B**. Paratype male (TL 258 mm) (ZVNU 110001); **C**. Female (TL 214 mm) (NMMB CD6300) [holotype of *B. paracelensis* Huang & Kawai, 2025]; **D**. Ovigerous female (TL 219 mm) (NMMB CD6299) [paratype of *B. paracelensis*]; **E**. Female (TL 217 mm) (NMMB CD6301) [paratype of *B. paracelensis*]; **F**. Ovigerous female (TL 210 mm) (NMMB CD6302) [paratype of *B. paracelensis*].

**Figure 2.**
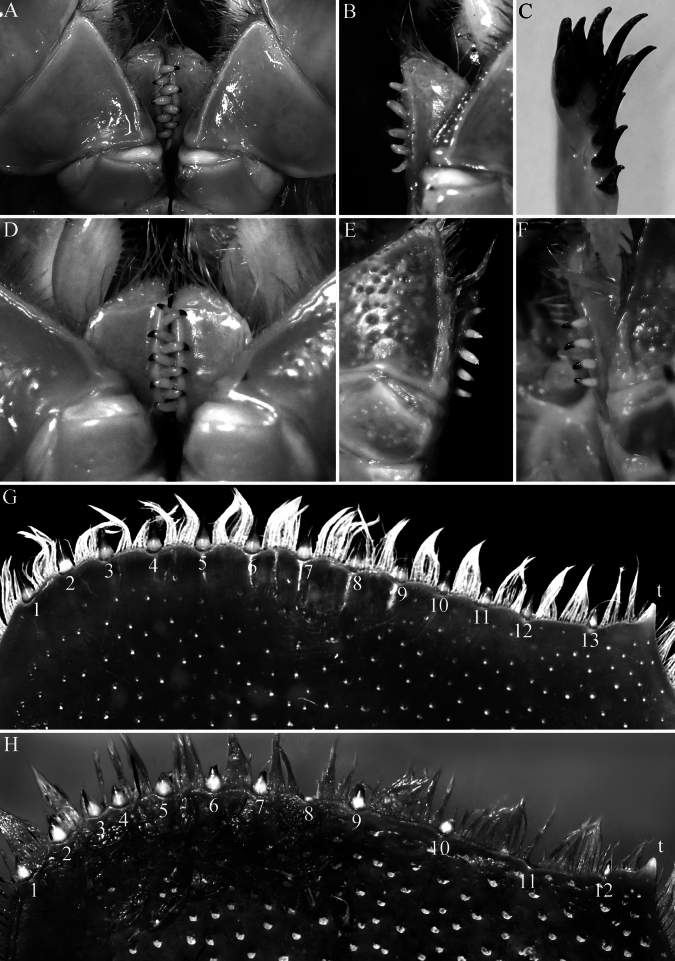
*Bathynomus vaderi* Ng, Sidabalok & Nguyen, 2025. **A–C, G**. Holotype male (TL 266 mm) (ZRC 2022.0621); **D**. Paratype male (TL 270 mm) (ZRC 2024.0176); **E, F, H**. Female (TL 214 mm) (NMMB CD6300) [holotype female of *Bathynomus paracelensis* Huang & Kawai, 2025]. **A, D**. Engaged closed endites of maxillipeds (right with 4 coupling hooks, left with 5 hooks); **B**. Left endite of maxilliped (with 5 hooks); **C**. Right maxillula with 11 keratinised spines; **E**. Right endite of maxilliped with 5 coupling hooks (detached); **F**. Left endite of maxilliped with 4 coupling hooks (in situ); **G**. Distal margin of left uropodal endopod (13 robust setae, all keratinous); **H**. Distal margin of left uropodal endopod (12 robust setae including damaged setae 8 and 10, seta 11 missing, remaining setae keratinised). Abbreviation: t = distolateral tooth of uropodal endopod.

**Figure 3.**
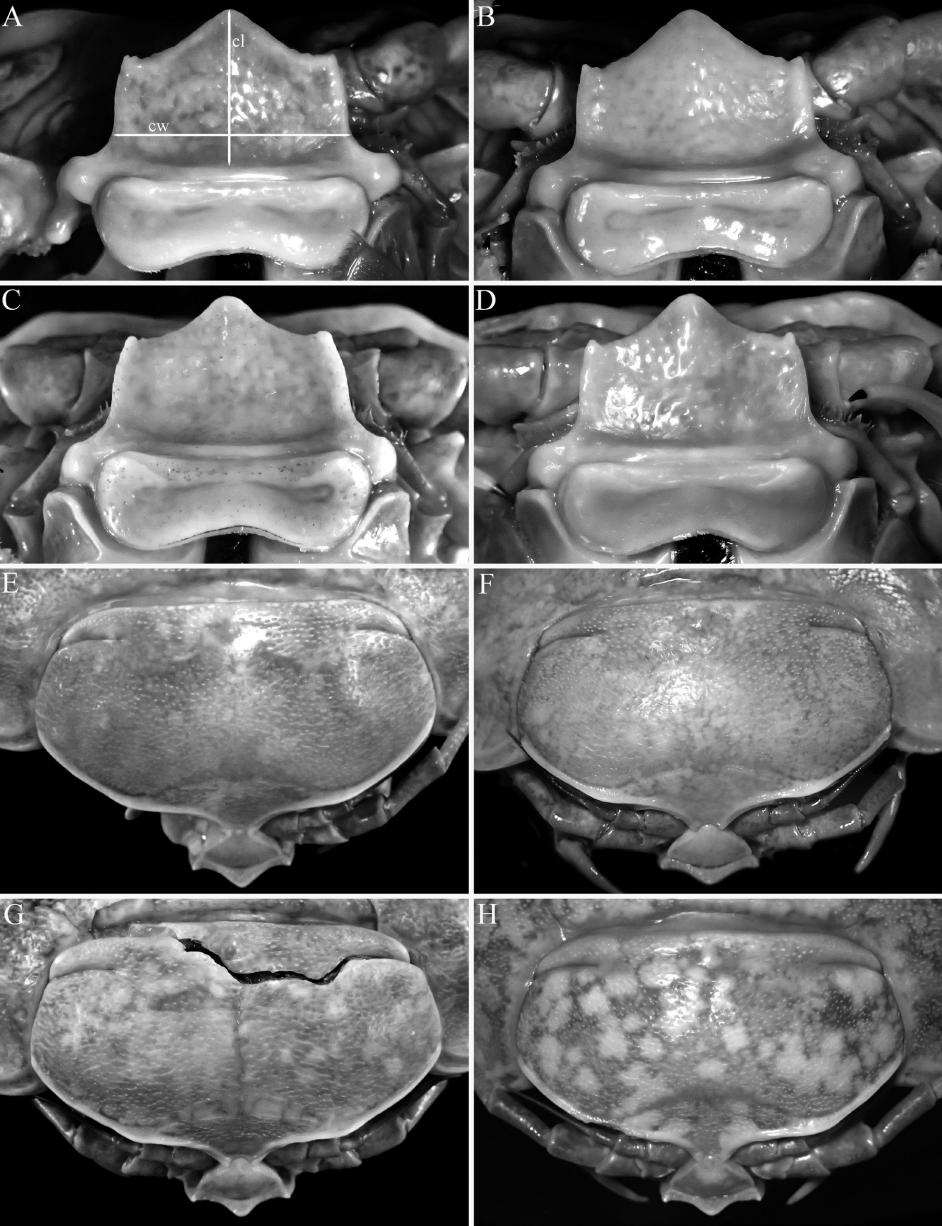
*Bathynomus vaderi* Ng, Sidabalok & Nguyen, 2025. **A–H**. Type specimens of *Bathynomus paracelensis* Huang & Kawai, 2025. **A, E**. Holotype female (TL 214 mm) (NMMB CD6300); **B, F**. Paratype ovigerous female (TL 219 mm) (NMMB CD6299); **C, G**. Paratype female (TL 217 mm) (NMMB CD6301); **D, H**. Paratype ovigerous female (TL 210 mm) (NMMB CD6302). **A–D**. Clypeus; **E–H**. Subdorsal view of cephalon. Abbreviations: cw = width of clypeus; cl = length of clypeus.

Tissue from 10 individuals of five species of *Bathynomus* (*Bathynomus* sp. as in Sidabalok et al. (2020), *B. affinis* Richardson, 1910, *B. jamesi*, *B. cf. jamesi*, and *B. vaderi*) were extracted for producing the novel sequences of two mitochondrial genes (COI and 16S rRNA) used in this study (Table 1). Crude genomic DNA was extracted from the muscles of the abdomen using the DNeasy^®^ Blood and Tissue Kit (Qiagen, Hilden, Germany) following the protocol of the manufacturer. Two primer sets, LCO1490/HCO2198 (Folmer et al. 1994) and Kmae/Kushi (Huang and Kawai 2025) were used for PCR amplification on COI gene, and16Sar/16S1472 (Simon et al. 1994; Crandall and Fitzpatrick 1996) for 16S gene. PCR reactions, cycling profiles, product checking, and sequencing procedures followed Yang et al. (2015). Output sequences were edited for contig assembly by SeqMan Pro^TM^ (Lasergene^®^; DNASTAR, Madison, WI, USA) before being blasted on GenBank (National Center for Biotechnology Information, NCBI) to check for any potential contamination. EditSeq (Lasergene^®^; DNASTAR) was used for translation into the corresponding amino acid sequences to avoid the inclusion of pseudogenes for the COI dataset.

**Table 1.** Specimens, localities and GenBank accession numbers of *Bathynomus* and outgroup species used in the present study.

Species	Voucher numbers	Locality	GenBank accession numbers	
			MtCOI	Mt16S rRNA
*B. affinis*	ZRC 2018.1069	Philippines	PZ044767*	PZ048044*
*Bathynomus* sp.	ZRC 2020.0017	Indonesia	PZ044768*	PZ048045*
*Bathynomus* sp.	MZB Cru.Iso 098	Indonesia	N.C.	PZ048046*
*B. doederleini* Ortmann, 1894	TC5	Taiwan	OQ421582	OQ915512
*B. giganteus*	HBG2706	Gulf of Mexico	MG229639	MG229479
*B. jamesi*	ZRC 2024.0118 (♂)	Vietnam	PZ044769*	PZ048047*
	ZRC 2024.0118 (♀)	Vietnam	PZ044770*	PZ048048*
	AM P109291	Vietnam	PZ044771*	N.C.
	HNO4	China	KX417647	KX417643
*B. kapala* Griffin, 1975	W29634	Australia	OQ970652	OQ971406
*B. kensleyi* Lowry & Dempsey, 2006	W29628	Australia	OQ860751	OQ865221
	W29629	Australia	OQ860752	OQ865222
	NTM Cr003425	Australia	OQ860753	N.C.
	W29630	Australia	OQ863731	OQ865220
*B. paracelensis*	NMMB-CD006299	South China Sea	PP715921	PP719187
	NMMB-CD006300	South China Sea	PP715922	PP719190
	NMMB-CD006301	South China Sea	PP715923	PP719189
	NMMB-CD006302	South China Sea	PP715924	PP719188
*B. cf. jamesi*	MNHN-IU-2013 6418	Philippines	PZ044772*	PZ048049*
*B. vaderi*	ZRC 2022.0621	Vietnam	PZ044773*	PZ048050*
	ZRC 2024.0176	Vietnam	PZ044774*	PZ048051*
	ZRC 2024.0180	Vietnam	PZ044775*	PZ048052*
*B. yucatanensis* Huang, Kawai & Bruce, 2022	YTNO1	Gulf of Mexico	MZ354630	MZ042927
**Outgroup**				
*Excirolana hirsuticauda*	–	–	MK917351	MK898194

* = New sequences resulted from this study. N.C. = No sequence available.

In addition to the sequences of two mitochondrial genes (COI and 16S rRNA) of *B. paracelensis*, other sequences of six congeners were downloaded from GenBank for the molecular analyses (Table 1). Sequence alignment was performed by the MAFFT v. 7 online service (Katoh et al. 2019) and a nucleotide length of 657 bp and 540 bp on COI and 16S rRNA genes, respectively, were used for the final analysed dataset. The gaps were filled by the fifth nucleotide “N”. Corrected pairwise distance was calculated based on the Kimura 2-parameter model (K2P; Kimura 1980) by MEGA v. 11 (Tamura et al. 2021). A maximum-likelihood (ML) tree was constructed using the IQ-TREE v. 3.0.1 (Wong et al. 2025) with 1,000 bootstrap replicates based on the model of “TIM2+G4+F” and with *Excirolana hirsuticauda* Menzies, 1962 used as an outgroup.

## Taxonomy

**Suborder Cymothoida Wägele, 1989**

**Family Cirolanidae Dana, 1852**

### Genus Bathynomus

Taxon classificationAnimaliaIsopodaCirolanidae

A. Milne-Edwards, 1879

E499D3F5-FE4E-5720-AB40-BEAD7182442D

#### Restricted synonymy.

See Lowry and Dempsey (2006: 168).

#### Type species.

*Bathynomus giganteus* A. Milne-Edwards, 1879; by monotypy.

### Bathynomus vaderi

Taxon classificationAnimaliaIsopodaCirolanidae

Ng, Sidabalok & Nguyen, 2025

E89F0BB2-7392-57D5-BC48-93F14D4E2514

Figs 1, 2, 3, 4

Bathynomus vaderi Ng, Sidabalok & Nguyen, 2025: 296, figs 4–8, 9A–D, 10A. — Ahyong 2025: 169, 170, 176, 177, 180, 181, fig. 8D. — Huang & Kawai, 2025: 2, 3, 15, 16, 18, 19, 21, 23, table 3.
Bathynomus paracelensis Huang & Kawai, 2025: 8, figs 1–6. [New synonymy].

#### Material examined.

*B. vaderi*—holotype: male (TL 266 mm) (ZRC 2022.0621), offshore of Quy Nhơn City, Bình Định Province (= Gia Lai Province), south-central Vietnam, South China Sea, ca. 50 nautical miles from shore, from deep-water (depth not known), Vietnam, purchased by Tran Anh Duc from Eo Gió, Nhơn Lý commune; 27 March 2022. Paratypes: 1 male (TL 270 mm) (ZRC 2024.0176), 1 male (TL 258 mm) (ZVNU 110001), 1 male (TL 257 mm) (ZVNU 110002), same data as holotype; 2 males (TL 325 mm, 295 mm) (ZRC 2024.0180), off Quảng Ngãi, Bình Định (= Gia Lai), Khánh Hòa and/or Phú Yên (Đắk Lắk) Provinces, central Vietnam, South China Sea, collected by trawlers, purchased by NTS from seafood restaurant in Đà Nẵng City, Vietnam, September 2024.

**Figure 4.**
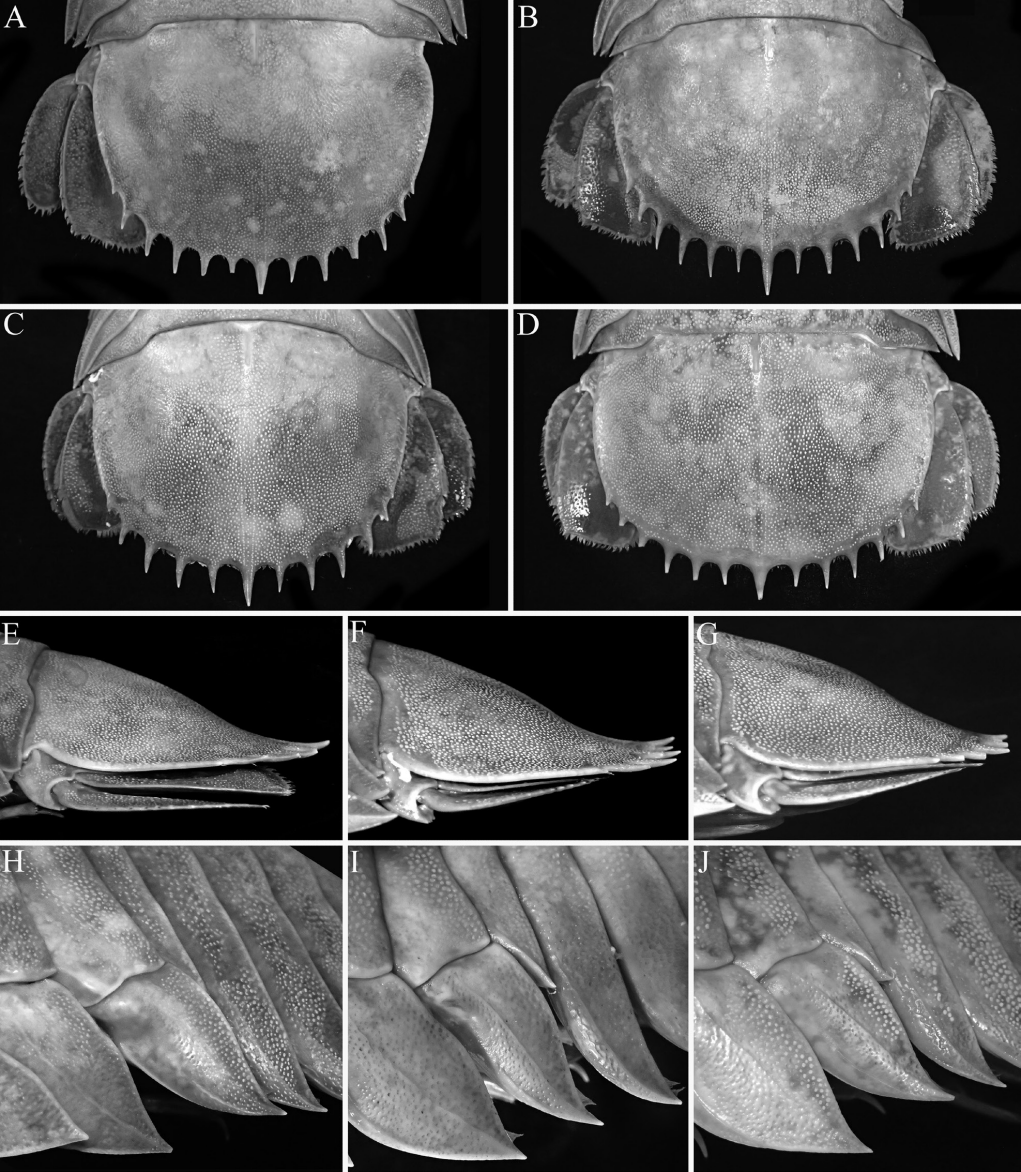
*Bathynomus vaderi* Ng, Sidabalok & Nguyen, 2025. **A–J**. Type specimens of *Bathynomus paracelensis* Huang & Kawai, 2025. **A, E, H**. Holotype female (TL 214 mm) (NMMB CD6300); **B**. Paratype ovigerous female (TL 219 mm) (NMMB CD6299); **C, F, I**. paratype female (TL 217 mm) (NMMB CD6301); **D, G, J**. Paratype ovigerous female (TL 210 mm) (NMMB CD6302). **A–D**. Dorsal view of pleotelson; **E–G**. Lateral view of pleotelson; **H–J**. Lateral view of pereon showing coxa of pereopod 7. Figure I laterally transposed to allow for comparisons.

*B. paracelensis*—holotype: female (TL 214 mm) (NMMB CD6300), Paracel Islands, Taiwan, South China Sea, 300–550 m, coll. bottom trawl, from fishermen by M.-C. Huang, 28 January 2023. Paratypes: 1 ovigerous female (TL 219 mm) (NMMB CD6299), 1 female (TL 217 mm) (NMMB CD6301), 1 ovigerous female (TL 210 mm) (NMMB CD6302), same data as holotype.

#### Comparative material.

*B. jamesi*—4 males, 3 females (NTOU), Tungsha Islands, coll. bottom trawl, from fishermen, provided by C.-H. Yang, 2023. Other material listed in Huang et al. (2022) and Ng et al. (2025) from MNHN, TMCD, ZRC and NTOU; and Ahyong (2025) from Australian Museum.

#### Remarks.

*Bathynomus vaderi* is best recognised by the following combination of characters: clypeus wide, broadly rectangular in ventral view, length to width ratio 0.62–0.70, lateral margins parallel, distal margins concave to almost straight, apex narrowly acute to gently rounded; pereopod 7 coxa proximally broad, distally narrowed and posteriorly curved; pleotelson with gently upwardly curved spines (almost straight in smaller specimens), with distinct row of setae present between spines; pleotelson 0.6 as long as wide; pleotelson longitudinally vaulted, dorsal margin in lateral view broadly convex; uropodal endopod distal margin with 11–15 (mean = 13) robust setae; lateral margin with continuous marginal setae along distal 83%, with 3–6 (mean = 4) robust setae; uropodal exopod length 2.5–2.6 × width, exopod distal margin with 4–7 (mean = 5) robust setae, lateral margin with 9–13 (mean = 11) robust setae; appendix masculina relatively short, reaching to posterior margin of pleopod 2 (updated from Ng et al. 2025).

Huang and Kawai (2025: 15, 16, table 3) compared *B. paracelensis* with *B. vaderi* and summarised 14 morphological characters that they note are different for *B. paracelensis*, in which 11 treated as diagnostic. These are now discussed below.

**Figure 5.**
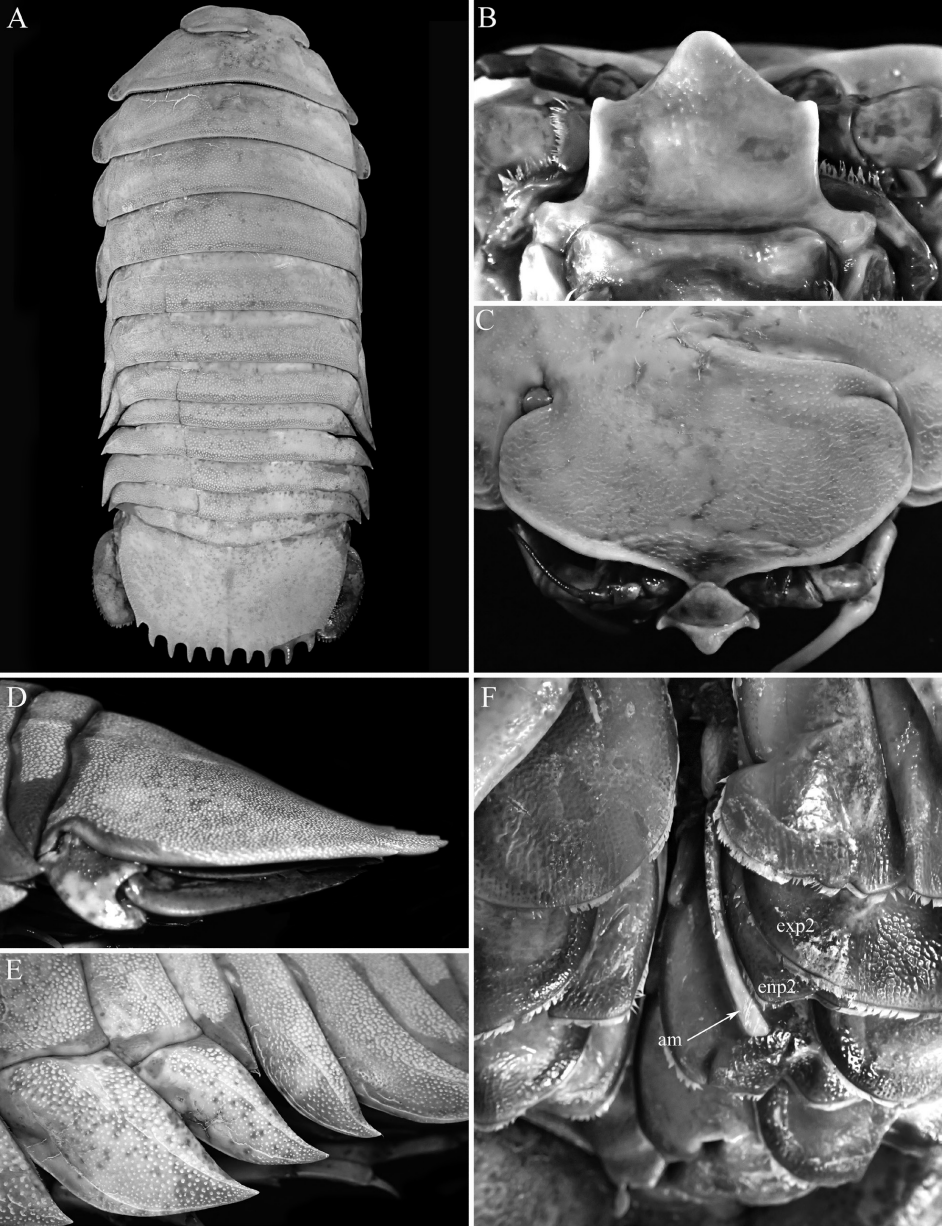
*Bathynomus jamesi* Kou, Chen & Li, 2017, male (TL 279 mm) (NTOU), Tungsha Islands. **A**. Dorsal habitus; **B**. Clypeus; **C**. Subdorsal view of cephalon (base of right side damaged); **D**. Lateral view of pleotelson; **E**. Lateral view of pereon showing coxa of pereopod 7; **F**. Length of left appendix masculina (right one missing). Abbreviations: am = appendix masculina; enp2 – endopod of pleopod 2; exp2 = exopod of pleopod 2.

**Figure 6.**
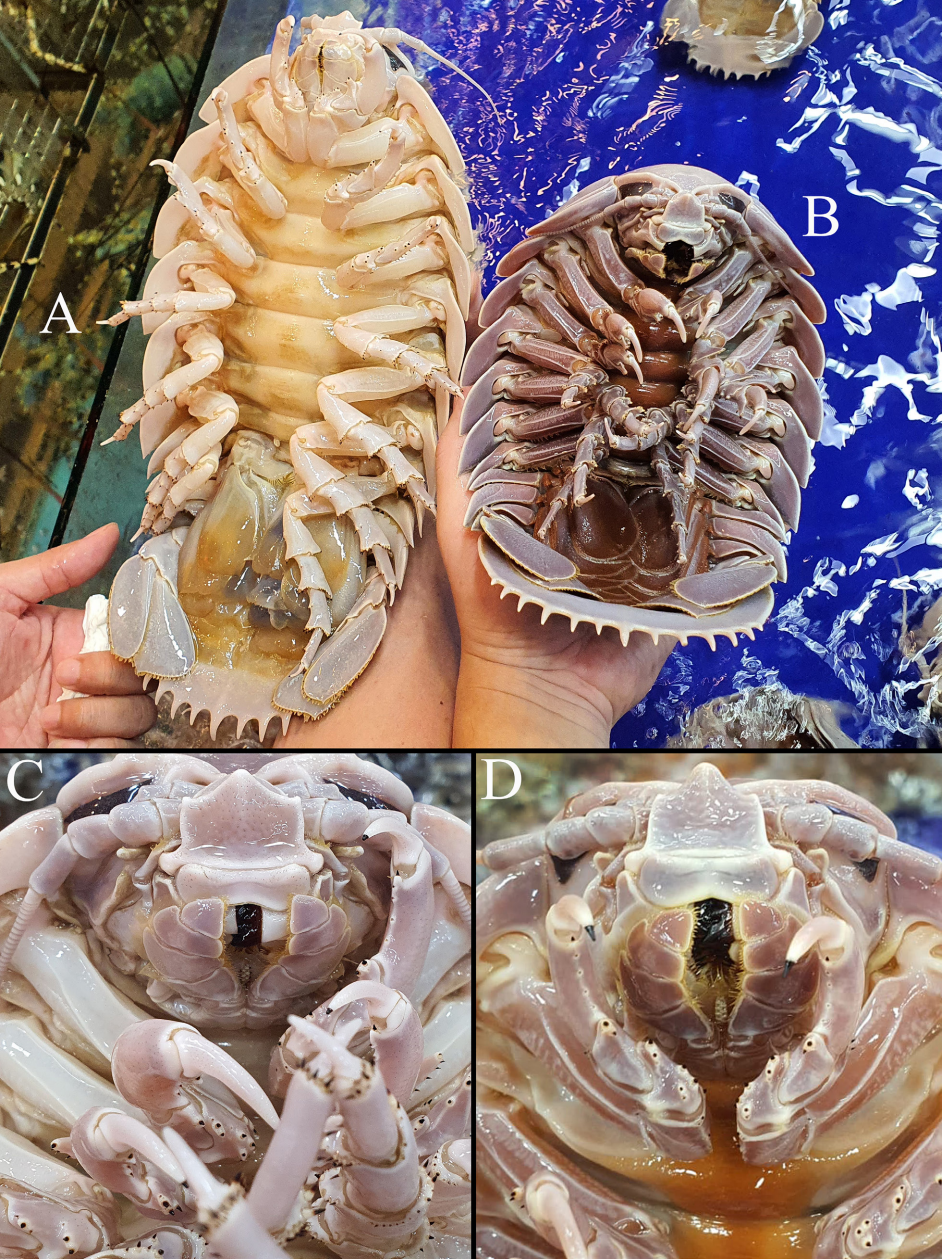
Colours in life. **A, C**. *Bathynomus vaderi* Ng, Sidabalok & Nguyen, 2025, paratype male (TL 325 mm) (ZRC 2024.0180); **B, D**. *B. jamesi* Kou, Chen & Li, 2017, male, not preserved. Both specimens from seafood centre in Đà Nẵng City, Vietnam, September 2024.

**Figure 7.**
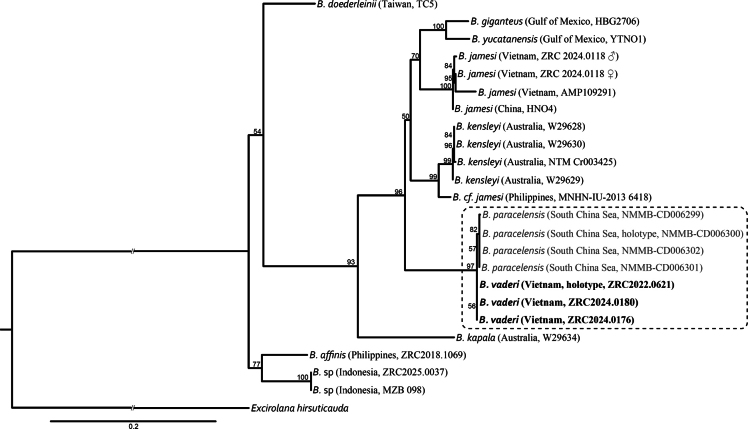
Maximum-likelihood tree (TIM2+G4+F model) among species of *Bathynomus* based on combined sequences of mitochondrial COI (657 bp) and 16S rRNA (540 bp) genes. *Excirolana hirsuticauda* is used as the outgroup. Numbers at nodes represent bootstrap support of 50% and above.

We here assess the characters cited by Huang and Kawai (2025) as diagnostic for *B. paracelensis* compared to those for *B. vaderi*:

1. ***Body length*** . All four female specimens of *B. paracelensis* (205–226 mm; mean 220 mm) are smaller than the six male specimens of *B. vaderi* (257–325 mm; mean 279 mm), but these differences are hardly compelling evidence for species distinctions given the small sample size and that the sexes are different. The sexual size difference is consistent with that observed in other supergiants in which males are typically larger than females (Briones-Fourzán and Lozano-Alvarez 1991; Soto and Mincarone 2001). The largest known specimens of *B. jamesi* are males, with females usually smaller (size range: for males 300–415 mm; for females 260–305 mm; Ng et al. 2025). Maximum size in cirolanids differs between species and should be measured separately for males, ovigerous females and non-ovigerous females where possible.
2. ***Body shape*** . Huang et al. (2022: 93) showed that body shape in *B. jamesi* (straight vs convex sided) can be variable in genetically identical specimens. In particular, the position of the coxal plates, which determines the dorsal outline of the pereon, can be affected by in-life effects, such as moulting and regeneration from damage, and post-mortem affects, such as compression/crushing during storage or preservation. There is also an inherent degree of flexion between the coxal plate and associated pereonite, and the degree of splaying of the coxal plates determines whether the body margins appear somewhat straight or more convex. Ng et al. (2025) studied 22 specimens of both sexes *B. jamesi* from Vietnam and Tungsha Islands, and observed that the body shape, i.e., whether the sides of the pereon are straighter or more convex is taxonomically unreliable and may be affected by many factors, including preservation. We have on hand another seven specimens from the Tungsha Islands, and they corroborate these observations. Our present molecular study also used specimens of *B. jamesi* of seemingly different body shape and their conspecificity is affirmed (Table 2, Fig. 7). The body of the type specimens of *B. paracelensis* does appear to be more slender than those of *B. vaderi*, but they are also less well preserved and had been frozen for long periods prior to preservation; in these specimens, the coxal plates appear to have been pushed inwards, possibly as a result of bulk storage in the freezer-hold of the fishing vessel, with the pereopods pulled inwards towards the body. In fact, we know from the fisherman who collected the type specimens for Huang and Kawai (2025) that they were frozen after capture, then passed to the authors for the study, thawed for their genetic and morphological work, before being frozen again by the authors. The four type specimens were presented to the curator of the NMMB in frozen state and he had to inject them with alcohol and preserve them for the museum. In the case of the type series of *B. vaderi*, the specimens were obtained alive, killed in ice water, and injected with 95% ethanol before being soaked in 80% ethanol for long-term preservation. It is evident from prior experience that repeated freezing and thawing can affect specimens’ shape and colour.
3. ***Colour of body and pereonite lateral margin*** . Ng et al. (2025) figured the colour of live or freshly dead specimens of *B. vaderi*; whereas those by Huang and Kawai (2025) are based on previously frozen specimens and those colours unequally compared and claimed as different by the latter. This is also the case with the colour differences observed for the lateral margin of the pereonite (white vs cream yellow). As such, the colour differences observed by Huang and Kawai (2025) are postmortem artefacts. Colour in life must be used judiciously in cirolanid taxonomy, given the high variability in many species, not to mention post-mortem changes following preservation, with the quality of preservative also affecting it. Although colour differences between *B. vaderi* and *B. paracelensis* are not supported here, it is nevertheless useful to document that because of the vibrant live seafood trade in Vietnam (see Ng et al. 2025), as two of the authors (TSN and PKLN) have observed hundreds of living *B. jamesi* and some *B. vaderi* kept alive in cold-water aquaria, and colour differences did exist between them. All the live *B. vaderi* we have observed have their sternum and pleopods pale yellow to cream (Fig. 6A, C; Ng et al. 2025: fig. 4B), whereas in *B. jamesi*, these structures are always purple to orange and brown (Fig. 6B, D; Ng et al. 2025: fig. 11B–D). The dorsal and lateral surfaces are more variable in colour, ranging from almost white to pale yellow and purple, and are thus unreliable as diagnostic characters. Once frozen or preserved, however, it is difficult to separate them based on colour.
4. ***Shape of the clypeus* (*anterior angle* )**. Huang and Kawai (2025: table 3) stated that the anterior angle of the clypeus is “narrowly round” (angle of 105°) for *B. paracelensis* and “narrowly subacute” (angle of 70°) for *B. vaderi*. A relatively sharper apex results in a more acute angle as well as more strongly concave margins on either side of the apex. The angle at which this structure is viewed is critical. The four type specimens of *B. paracelensis* have more rounded apices (Fig. 3A–D) than the holotype of *B. vaderi* (Ng et al. 2025: figs 5C, 6C, 9A) but it actually agrees very well with a paratype of the latter (Ng et al. 2025: fig. 4C). As such, the differences observed between *B. paracelensis* and *B. vaderi* are within the normal range of variation for the species. The form of the apex of the clypeus also varies in the large series of specimens of *B. jamesi* studied herein and the type series of *B. wilsoni* (Ahyong 2025: figs 2B, 6B). The broadly rectangular clypeus is one of the diagnostic features of *B. vaderi*. The clypeus of one recent male of *B. jamesi* is clearly more rectangular (Fig. 5B) than that of typical specimens which is normally distinctly more quadrate (see Ng et al. 2025). The clypeus of this specimen (Fig. 5B) superficially resembles that of *B. vaderi*, but when compared directly, it is still proportionately narrower (Fig. 3A–D).
5. ***Number of coupling hooks on maxilliped endite*** . The number of coupling hooks is mostly uninformative between closely related species of *Bathynomus*. Most cirolanids have two (sometimes 1 or 3) and *Eurydice* Leach, 1815 is without coupling hooks, while species of *Bathynomus* have 4–7 coupling hooks (4 or 5 in supergiants) (Bruce 1986). Huang and Kawai (2025) stated that *B. paracelensis* has five hooks; but on the holotype female (NMMB CD6300), while the right endite has five hooks (Fig. 3E), the left endite has only four hooks (Fig. 3F). This is also the case for the holotype male of *B. vaderi* where the right endite has four hooks while the left has five (Fig. 3A, B). The other paratype males have either four or five hooks on the endites (Fig. 3D). In the paratype females of *B. paracelensis*, the number of hooks on the left and right endites, respectively, are: 4/4 (NMMB CD6299), 4/4 (NMMB CD6301), and 5/5 (NMMB CD6302). As such the number of coupling hooks varies even in the type series of *B. paracelensis*. It must be noted that coupling hooks often get dislodged, so the absence of a hook is not atypical, and within the Cirolanidae, the presence of an “additional” coupling hook is also not unusual.
6. ***Number of keratinised robust setae on maxillula*** . This is not a taxonomically useful character, and the form of the serration and structure of the spines is probably more useful than the number present. In addition, the number and pattern of these robust setae scarcely differ at the generic level throughout the Cirolanidae. In any case, these spines are difficult to count in lateral view, and one needs an “en face” view to be sure that none of the small ones are hidden, and as such is often inaccurately observed or described. A re-examination of the holotype male of *B. vaderi* shows two smaller spines behind those figured (Ng et al. 2025: fig. 7E), so there are in fact 11 spines present (Fig. 2C). All the other paratype males have 11 spines on the maxillula so the two species do not differ. There is some variation in the number of these spines on the maxillula. The holotype female of *B. paracelensis* (NMMB CD6300) has both maxillulae armed with 11 spines. In the paratype females, the number of spines on the left and right maxillulae, respectively, are 10/10 (NMMB CD6299), 10/right missing (NMMB CD6301), and 10/10 (NMMB CD6302).
7. ***Pleotelson length to width ratio*** . The proportions of the pleotelson do not separate *B. paracelensis* from *B. vaderi*—even the original proportions provided by Huang and Kawai (2025) overlap (0.51–0.62 vs 0.6) (Fig. 4A–D). Our own measurements of the four female types of *B. paracelensis* and six male types of *B. vaderi* do not differ.
8. ***Pleotelson spine number*** . The number of spines is diagnostically important, but a variation of one or two over a range of 10–12 is within the range of intraspecific variation observed in other supergiants (Lowry and Dempsey 2006; Ahyong 2025). The count for *B. paracelensis* was given as 12 or 13 and for *B. vaderi* it is 11+2. This “difference” is merely notational and not actual because Ng et al. (2025) treated the smallest lateral spine separately from the larger ones, whereas Huang and Kawai (2025) counted all of them together.
9. ***Pleotelson spine structure*** . The shape of the spines is taxonomically important and useful to separate species. The two largest female type specimens of *B. paracelensis* (219 mm, NMMB CD6299; 217 mm, NMMB CD6301) have pleotelson spines slightly curving upwards (Fig. 4F), whereas these spines in the smaller holotype (NMMB CD6300) and third paratype female (NMMB CD6302) are more weakly curved (Fig. 4E, G). The type specimens of *B. vaderi* are all larger than those of *B. paracelensis* and, thus, have more strongly curved spines. This is already known for *B. jamesi* (and other species) where the degree of curvature is size-related (see Ng et al. 2025).
10. ***Robust setae on uropodal endopod distal margin*** . The total number of robust setae on the distal margin of males and females is 12 or 13, but there does appear to be slight differences in how they are arranged. In the female specimens of *B. paracelensis* examined, the robust setae on the distal margin are relatively larger and more closely spaced just after the distomedial angle, becoming smaller and more widely spaced as the margin curves inwards (Fig. 2G; Huang and Kawai 2025: fig. 3C, D). These setal insertions on the article margin can be readily discerned even when the setae are damaged or broken off completely (Fig. 2G). In the holotype female (NMMB CD6300), the robust setae observed on the outer part of the distal margin are apically keratinised, so they are very obvious, whereas those on the inner one-fifth of the margin appear unarmed (when superficially examined), despite there actually being short and soft non-keratinised spines (Fig. 2G). This is also the case for the other paratypes, although one specimen (NMMB CD6301) has the entire left distal margin armed with only a few low non-keratinised setae, but that is evidently abnormal. In the male specimens of *B. vaderi*, all the robust setae on the distal margins are keratinised (Fig. 2F), but in one paratype male (TL 295 mm, ZRC 2024.0180), the inner fifth part of the left and right distal margins also appear unarmed, with only low non-keratinised setae present.
11. ***Uropodal exopod distolateral angle*** . Huang and Kawai (2025) stated that the distolateral angle of the uropodal exopod of *B. paracelensis* is slightly produced (vs produced in *B. vaderi*) but we cannot discern any significant differences—they are all produced and acute, as is the case for several species of supergiant *Bathynomus*.

The present morphological comparisons of the type specimens of *B. vaderi* and *B. paracelensis* show that the purported differences between the two species do not stand up to scrutiny. All the morphological differences noted by Huang and Kawai (2025) to separate the two species are shown to be due to intraspecific variation, inaccurate observation, and/or accounted for by condition and state of preservation of the specimens. We cannot morphologically distinguish the two nominal species, with all the diagnostic characters for *B. paracelensis* shared with *B. vaderi*. We also analysed the molecular relationship between the two species. Sequences of mitochondrial COI and 16S rRNA genes were compared: the divergences of these two genes between *B. vaderi* and *B. paracelensis* are all less than 1% (COI: 0; 16S: 0.6–0.8%). The variance is at the intraspecific level, as shown by the divergences among individuals of *Bathynomus* sp., *B. jamesi*, *B. kensleyi*, *B. paracelensis* and *B. vaderi* (Table 2). This similarity is also reflected on the ML tree in which *B. vaderi* and *B. paracelensis* form a stable clade with high support (BS = 97) (Fig. 7).

**Table 2.** Pairwise distance (%) based on the Kimura 2-parameter (K2P) model of mitochondrial COI (under diagonal shade, 657 bp) and 16S rRNA (above diagonal shade, 540 bp) genes among species of *Bathynomus* and the outgroup. Numbers in the square brackets are sample size. The divergence percentages between *B. paracelensis* and *B. vaderi* are shown in bold.

	1	2	3	4	5	6	7	8	9	10	11	12
1- *B. affinis* (Philippines)	*	6.9–7.2	12.0	17.6	16.7–17.5	18.1	17.6–18.0	20.2–20.6	15.2	18.9	17.8	63.1
2-*Bathynomus* sp. (Indonesia) [2]	15.1	N.C. (COI);<br/> 0 (16S)	10.7–11.4	16.7–17.2	16.4–20.0	16.1–16.8	17.3–18.0	19.5–19.9	17.5–19.4	18.3–20.1	16.8–16.9	59.6–61.0
3-*B. doederleini*	12.7	14.2	*	15.1	15.5–17.2	15.4	13.8–14.1	17.0–17.4	18.6	18.1	14.3	67.2
4-*B. giganteus*	22.9	23.4	23.3	*	4.8–5.7	14.3	6.8–7.1	8.5–8.7	7.1	7.7	3.7	68.9
5- *B. jamesi* [4]	25.2–25.6	27.4–2810	26.6–27.2	12.9–13.2	0–0.5 (COI); 0.4–0.5 (16S)	13.6–15.2	6.1–6.8	8.8–10.4	5.7–6.4	8.0–9.0	4.9–5.3	66.9–81.3
6-*B. kapala*	23.8	25.4	25.7	20.4	20.4–20.9	*	11.9–12.2	15.1–15.4	13.5	16.2	11.0	66.9
7-*B. kensleyi* [4]	25.4–28.6	26.3–29.2	25.4–30.0	14.2–15.4	10.8–12.0	18.8–20.7	0–0.5 (COI); 0–0.2 (16S)	9.2–9.7	3.0	9.8–10.1	4.4–4.6	65.9
8- *B. paracelensis* [4]	23.0	24.4	26.4	16.2	17.1–17.7	24.3	15.7–16.8	0 (COI); 0–0.2 (16S)	13.2–13.7	0.6–0.8	8.8–9.1	64.7–65.6
9- *B. *cf. j*amesi*	24.6	25.5	25.1	14.1	10.8–11.2	19.6	3.7–4.6	15.4	*	11.6	5.3	75.7
10-*B. vaderi* [3]	23.5	24.9	25.4	16.2	16.5–17.1	24.1	15.1–16.5	0.0000	15.4	0 (COI & 16S)	8.9	64.8
11-*B. yucatanensis*	23.5	25.0	22.6	6.5	13.6–14.0	20.4	14.0–16.0	18.7	14.5	18.4	*	67.7
12-*Excirolana hirsuticauda*	38.3	37.6	39.5	42.0	43.4–44.4	38.0	45.7–47.0	39.8	45.2	38.5	43.9	*

The conclusion that *B. paracelensis* and *B. vaderi* are conspecific is inescapable. Although both species were described in the same year, *B. vaderi* has nomenclatural priority, with Ng et al. (2025) published on 14 January 2025 whereas Huang and Kawai (2025) was published on 31 March 2025. As such, *Bathynomus paracelensis* Huang & Kawai, 2025, is herein synonymised with *Bathynomus vaderi* Ng, Sidabalok & Nguyen, 2025. Nevertheless, Huang and Kawai (2025) provided the first documentation of adult females of *B. vaderi*.

Much work still needs to be done with *Bathynomus* taxonomy, with more species still to be discovered. Many recent workers follow the standard and format in the important revision of the genus by Lowry and Dempsey (2006), who under editorial pressure, gave only diagnoses of their species. There is a need for more complete descriptions and more figures for species of *Bathynomus*, especially because new characters are being continually found that help distinguish species. For example, the appendix masculina, when possible, should be described and figured (Ng et al. 2025), the length: width ratio of the uropodal exopod in adults, and the relative positions of the pleural apices of the posterior pleonites carefully observed (Ahyong 2025). The appendix masculina is highly likely to differentiate males of similar sympatric species (e.g., between *B. vaderi* and *B. jamesi*) but has rarely been documented in the past. Equally important is for the range of character variation to be documented. It is essential that perceived “outliers” are not excluded. This variation includes size and should be recorded separately for the sexes. For species of *Bathynomus* it is important to record the range and median number of uropodal robust setae (with separate totals for the different margins) and the number, shape, and development of the pleotelson spines as well. As in the case of *B. paracelensis* here, we emphasise that variation in a purported species needs to be fully documented, and the characters must not be “cherry picked” to support a preferred outcome.

## Supplementary Material

XML Treatment for Bathynomus

XML Treatment for Bathynomus vaderi

## Additional information

### Use of AI

No use of AI was reported.

### Author ORCIDs

Peter K. L. Ng https://orcid.org/0000-0001-5946-0608

Niel L. Bruce https://orcid.org/0000-0003-4745-5048

Conni M. Sidabalok https://orcid.org/0000-0002-4325-0558

Chien-Hui Yang https://orcid.org/0000-0002-4594-3622

Thanh Son Nguyen https://orcid.org/0000-0001-5260-5536

Shane T. Ahyong https://orcid.org/0000-0002-2820-4158

### Data availability

All of the data that support the findings of this study are available in the main text or Supplementary Information.
